# *Bacopa monnieri* phytochemicals regulate fibroblast cell migration via modulation of focal adhesions

**DOI:** 10.1016/j.isci.2024.109489

**Published:** 2024-03-11

**Authors:** Ravindra K. Zirmire, Dyuti Saha, Rakesh Dey, Habibu Tanimu, Rania Zaarour, Deborah Bird, Prakash Cherian, Isha Rana, Nita Roy, Aryasekhar Sanyal, Namita Misra, Colin Jamora

**Affiliations:** 1 IFOM-inStem Joint Research Laboratory, Centre for Inflammation and Tissue Homeostasis, Institute for Stem Cell Science and Regenerative Medicine (inStem), Bangalore, Karnataka 560065, India; 2 Shanmugha Arts, Science, Technology and Research Academy (SASTRA) University, Thanjavur, Tamil Nadu 613401, India; 3 Department of Biology, Manipal Academy of Higher Education, Manipal, Karnataka, India; 4 JAIN (Deemed-to-be University), #44/4, District Fund Road, Jayanagar 9th Block, Bangalore, Karnataka 560069, India; 5 Thumbay Research Institute for Precision Medicine, Gulf Medical University, Ajman, UAE; 6 L’Oréal, Research and Innovation, Aulnay, France; 7 L'Oréal, Research & Innovation, Bengaluru, India

**Keywords:** Phytopharmacy, Molecular biology, Cell biology, Phytochemistry

## Abstract

The *Bacopa monnieri* plant contains phytochemicals that have been used extensively in traditional medicine to treat various diseases. More recently it has been shown to accelerate wound healing, though its mechanism of action is largely unknown. Here we investigated the cellular pathways activated by a methanol extract of *Bacopa monnieri* in human dermal fibroblasts, which play many critical roles in the wound healing program. Gene expression analysis revealed that the *Bacopa monnieri* extract can modulate multiple processes involved in the wound healing program such as migration, proliferation, and angiogenesis. We discovered that the extract can increase migration of fibroblasts via modulating the size and number of focal adhesions. *Bacopa monnieri*-mediated changes in focal adhesions are dependent on α5β1 integrin activation and subsequent phosphorylation of focal adhesion kinase (FAK). Altogether our results suggest that *Bacopa monnieri* extract could enhance the wound healing rate via modulating fibroblast migration into the wound bed.

## Highlights

• *Bacopa monnieri* extract accelerates wound healing but the mechanism remains unknown
• Phytochemicals from *Bacopa monnieri* can stimulate fibroblast migration
• The pro-migratory effect of the extract is through the modulation of focal contacts
• *Bacopa monnieri* extract also impacts proliferation and angiogenesis

## Graphical abstract

**Figure:**
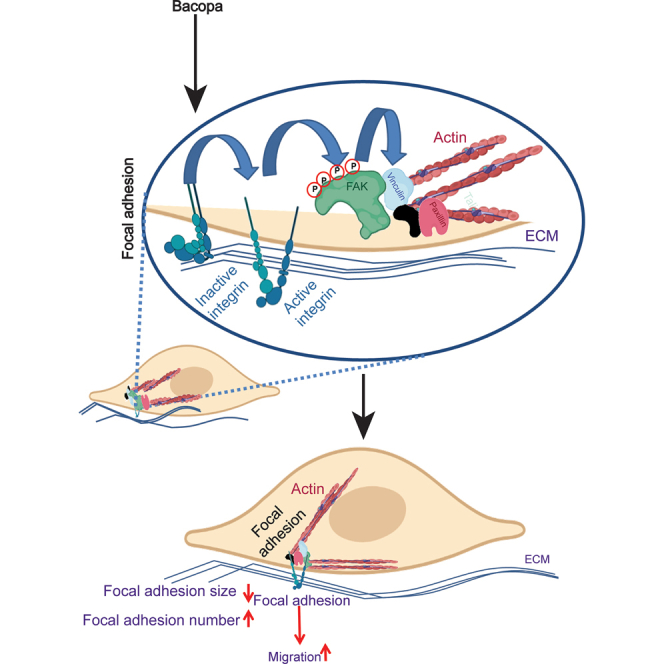

## Introduction

Wound healing comprises three distinct but overlapping phases. The initial phase is the inflammatory phase marked by the activation of resident immune cells and infiltration of circulating immune cells. The second phase is the proliferative phase wherein the proliferation and migration of fibroblasts, keratinocytes, and stem cells occur, in addition to the secretion of extracellular matrix (ECM) and angiogenesis. Finally, the wound-healing program culminates in the remodeling phase which comprises the removal of excess ECM and restructuring of cell-cell and cell-matrix interactions. Impairment of any of these phases can lead to delays in the kinetics of the wound-healing program. The phases of the wound healing program are the result of extensive and carefully orchestrated intercellular crosstalk. A major regulatory node of this intercellular network is the dermal fibroblasts that have been shown to impact all the phases of the wound healing program such as inflammation,^1^ angiogenesis,^2^ stem cell proliferation, and migration^3^^,^^4^ and protease release for tissue remodeling.^5^

However, in various disease contexts, prolonged inflammation can lead to fibroblast senescence^6^ and reduced wound healing activities. As a consequence of crippling fibroblast activity, the wound-healing program is significantly impaired. Thus, one method of restoring wound healing in chronic inflammatory conditions such as diabetes would be to repair fibroblast dysfunction. Herbal remedies have been proposed as a method to stimulate proliferation and differentiation of human mesenchymal stromal cells for cell therapies^7^ and we investigated whether this same approach can be utilized for the promotion of fibroblast wound-healing activities. Extracts of *Bacopa monnieri* have been used in traditional medicine for the treatment of multiple diseases including Alzheimer’s disease,^8^ epilepsy,^9^ and anxiety and depression.^10^ In addition, it has been used for memory enhancement,^11^ as a nootropic agent,^12^ and interestingly for the enhancement of wound healing.^13^

It has been shown that *Bacopa monnieri* extract, when administered orally, increases the wound closure rate in rats through its antioxidant activity.^13^^,^^14^ It increased the levels of antioxidants such as Glutathion (GSH), superoxide dismutases (SOD), and catalase (CAT), whereas it reduced the levels of free radicals Lipid peroxidation (LPO) and nitric oxide (NO).^13^ Oxidants and free radicals are regulated by the inflammatory environment during the wound healing process.^15^ There was also a significant reduction in the inflammatory cells as well as inflammatory markers, increased neovascularization,^13^ and increased re-epithelialization.^14^ Altogether these results suggest that *Bacopa monnieri* extract affects inflammatory cells, endothelial cells, and keratinocytes to accelerate the wound healing process though the mechanisms and signaling pathways mediating these effects remain unknown. Given the importance of dermal fibroblasts in the cutaneous wound healing response, we investigated whether *Bacopa monnieri* extract can modulate the wound-healing activities of human fibroblasts and the mechanism of its action.

## Results

### *Bacopa monnieri* extract increases fibroblast migration

**Figure 1:**
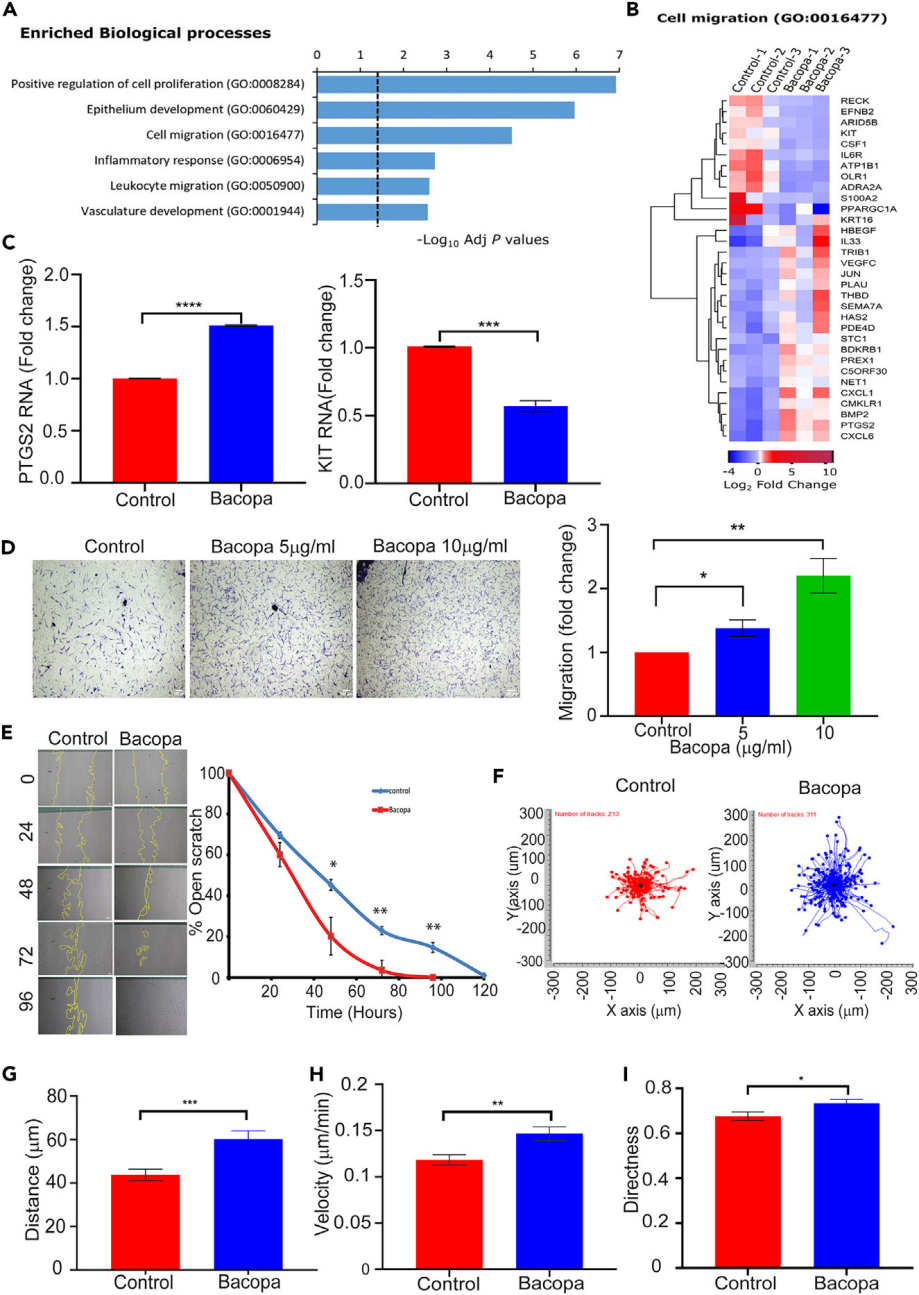
*Bacopa monnieri* extract increases human dermal fibroblast migration (A) Enriched biological processes after RNA sequencing analysis of fibroblasts treated with *Bacopa monnieri* extract. (B) Heatmap of genes associated with cell migration in cells treated with *Bacopa monnieri* or vehicle control (water). (C) qPCR analysis of PTGS2 and KIT gene expression. (D) Transwell migration of cells in the presence of *Bacopa monnieri* or vehicle control was analyzed by crystal violet staining (left panel) and quantified (right panel). Scale bar: 100 μm. (E) Scratch migration of cells in the presence of Bacopa or vehicle control (left panel) and quantification (right panel), scale bar: 50 μm. p values were calculated using two-way ANOVA followed by post hoc Šídák’s multiple comparisons test. ∗p ≤ 0.05, ∗∗p < 0.01, ∗∗∗p < 0.001, ∗∗∗∗p < 0.0001, (n = 3 biological replicates). (F) Quantification of 2D random migration of fibroblasts treated with *Bacopa monnieri* extract or vehicle control. (G–I) Analysis of distance (G), velocity (H), and directness (I) of cell migration of individual fibroblasts in the presence of *Bacopa monnieri* extract (n = 311 cells) or vehicle control (n = 213 cells). p values were calculated using unpaired Student’s t test. ∗p ≤ 0.05, ∗∗p < 0.01, ∗∗∗p < 0.001.

To test the effect of *Bacopa monnieri* extract on primary human dermal fibroblasts, we first determined the maximal tolerable concentration by measuring cell viability in the presence of the extract (Figure S1A). The maximum tolerable concentration was found to be 10 μg/mL. To take an unbiased approach to decipher the effect of *Bacopa monnieri* extract, RNA sequencing analysis was performed on adult fibroblasts treated with *Bacopa monnieri* extract for 12 h. Interestingly, gene cluster analysis revealed that *Bacopa monnieri* extract modulated several genes associated with multiple processes of the wound-healing program (Figure 1A). Though the positive regulation of cell proliferation was the top biological process, *Bacopa monnieri* extract-treated fibroblasts did not display an increase (Figure S1B) or decrease (Figure S1C) in the proliferation rate compared to control-treated cells.

On the other hand, a major biological process that was deemed to be affected by *Bacopa monnieri* exposure is cell migration (Figures 1A and 1B). For instance, quantitative PCR confirmed that the pro-migratory gene PTGS2^16^ is upregulated, while the migration inhibiting gene ADRA2A^17^ is downregulated (Figure 1C). To determine whether the changes in gene expression are manifested in the biological activity of the dermal fibroblasts, we performed a transwell migration assay. Primary human dermal fibroblasts were seeded in the top compartment of the transwell membrane and *Bacopa monnieri* extract or vehicle control was added to the media of the lower compartment. 12 h post-incubation, cells were fixed and the cells which had migrated from the top of the transwell to the bottom were counted. Interestingly, it was observed that *Bacopa monnieri* extract increased the chemotactic migration of fibroblast in a concentration-dependent manner (Figure 1D). In order to test if *Bacopa monnieri* extract can increase migration in a two-dimensional migration platform, a scratch wound assay was performed. Cells were cultured to form a confluent sheet and treated with mitomycin C to block proliferation. A “wound” was induced by using a pipet tip to scratch the cells and form a gap in the cell sheet. The scratch wound was monitored for the rate of closure by imaging and quantifying the wound area every 24 h. It was observed that *Bacopa monnieri* extract substantially increased the wound closure rate of cells (Figure 1E) in a concentration dependent manner (Figure S1D). Since *Bacopa monnieri* extract increased the directional migration of cells in both a 3D and 2D assay, we investigated whether it also promoted the general motility of the fibroblast. Analysis of random migration was performed by the live tracking of the nuclear location of individual, underconfluent cells with brightfield microscopy in the presence of *Bacopa monnieri* extract or vehicle control for 12 h (Figure 1F). The motility analysis of single cells was performed with the ImageJ manual tracker and revealed that *Bacopa monnieri* extract significantly increased the average distance moved by the cell from its starting point at the beginning of the analysis (Figure 1G). In addition, the velocity of the *Bacopa monnieri* treated cells was elevated relative to fibroblasts treated with vehicle control (Figure 1H). Interestingly, it was observed that *Bacopa monnieri* extract also increased the directionality of the migration (Figure 1I). Overall, these data indicate that *Bacopa monnieri* extract increased the basic motility of the fibroblasts as well as their directional migration.

### *Bacopa monnieri* extract treatment modulates focal adhesions in human dermal fibroblasts

**Figure 2:**
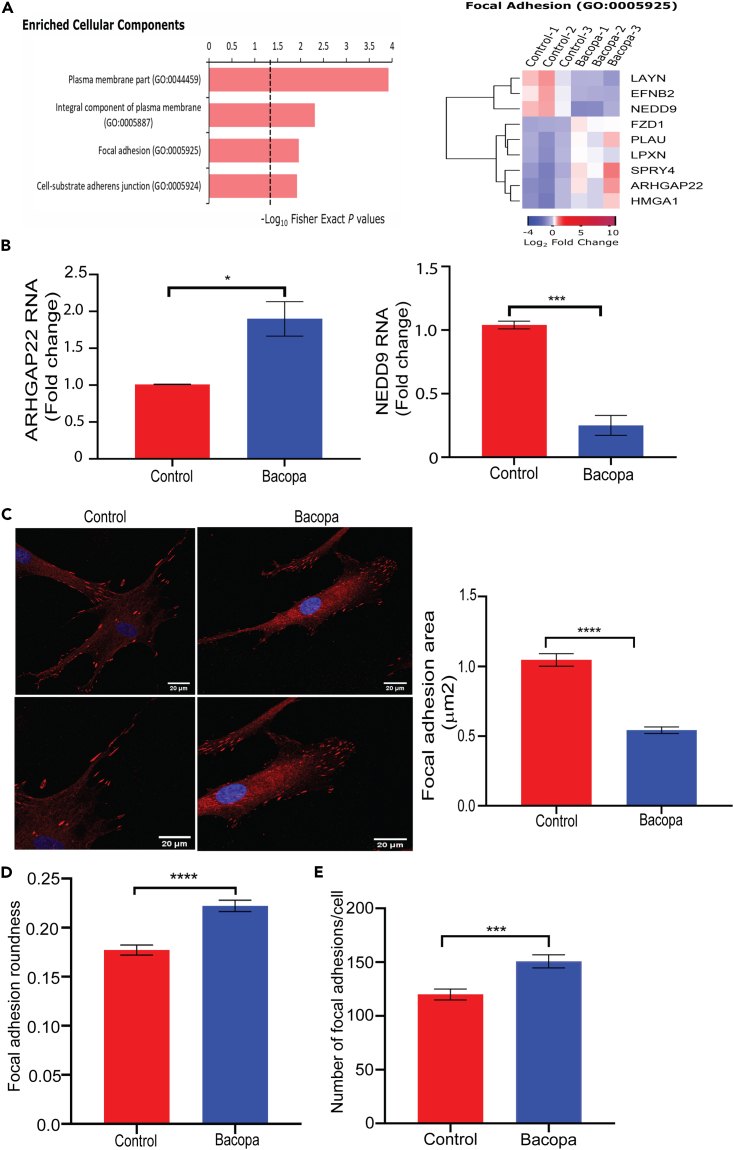
*Bacopa monnieri* extract alters focal adhesions in fibroblasts (A) Cellular component analysis from RNA sequencing of fibroblasts treated with *Bacopa monnieri* extract (left panel). Heatmap of genes associated with focal adhesions (right panel). (B) qPCR analysis of ARHGAP22 and NEDD9 gene expression. (C) Vinculin staining (red) in human dermal fibroblast treated with *Bacopa monnieri* extract or vehicle control, DAPI (blue) marks nuclei (left panel) and quantification of focal adhesion area (right panel). Scale bar 20 μm. (D and E) Analysis of focal adhesions roundness (D) and number per cell (E). p values were calculated using unpaired Student’s t test. ∗p ≤ 0.05, ∗∗p < 0.01, ∗∗∗p < 0.001, vehicle control n = 138 cells, *Bacopa monnieri* extract n = 144 cells.

To elucidate the mechanism by which *Bacopa monnieri* extract increases human dermal fibroblast migration, the transcriptome profile was analyzed for enriched cellular components. Interestingly, it was observed that the *Bacopa monnieri* extract modulated several genes associated with cell substratum adhesion and genes associated with structural components of focal adhesions (Figure 2A). We confirmed the changes in focal adhesion associated genes by RT-PCR and it validated the upregulation of ARHGAP22, a member of the RhoGAP family of proteins with pro-migratory effects^18^ and downregulation of NEDD9, which stabilizes focal contacts and suppresses migration^19^ (Figure 2B). The focus on focal adhesions is particularly relevant since their size and shape have been shown to uniquely predict the rate of cell migration^20^^,^^21^ To assess if *Bacopa monnieri* extract also affects the size of focal adhesions, vinculin (a structural component of focal adhesions) was characterized as a proxy for focal adhesion morphology. The size and shape of focal adhesions were quantified using ImageJ manual measuring with the “freehand selection” tool. Interestingly, it was observed that the *Bacopa monnieri* extract-treated fibroblasts exhibited reduced focal adhesion size throughout the cell (Figure 2C), and an altered shape from elongated to a more spherical morphology (Figure 2D) in a concentration dependent manner (Figure S2A). Consistent with the changes in the size and shape of the focal adhesions, treatment of cells with Bacopa extract led to a reduction in the size (Figure S2B) and increase in the roundness of the cell (Figure S2C). Additionally, it was found that the decreased size and increased roundness of the focal adhesions in *Bacopa monnieri* extract-treated cells were compensated by an increased number of focal adhesions (Figure 2E).

### *Bacopa monnieri* extract activates α5β1 integrin

**Figure 3:**
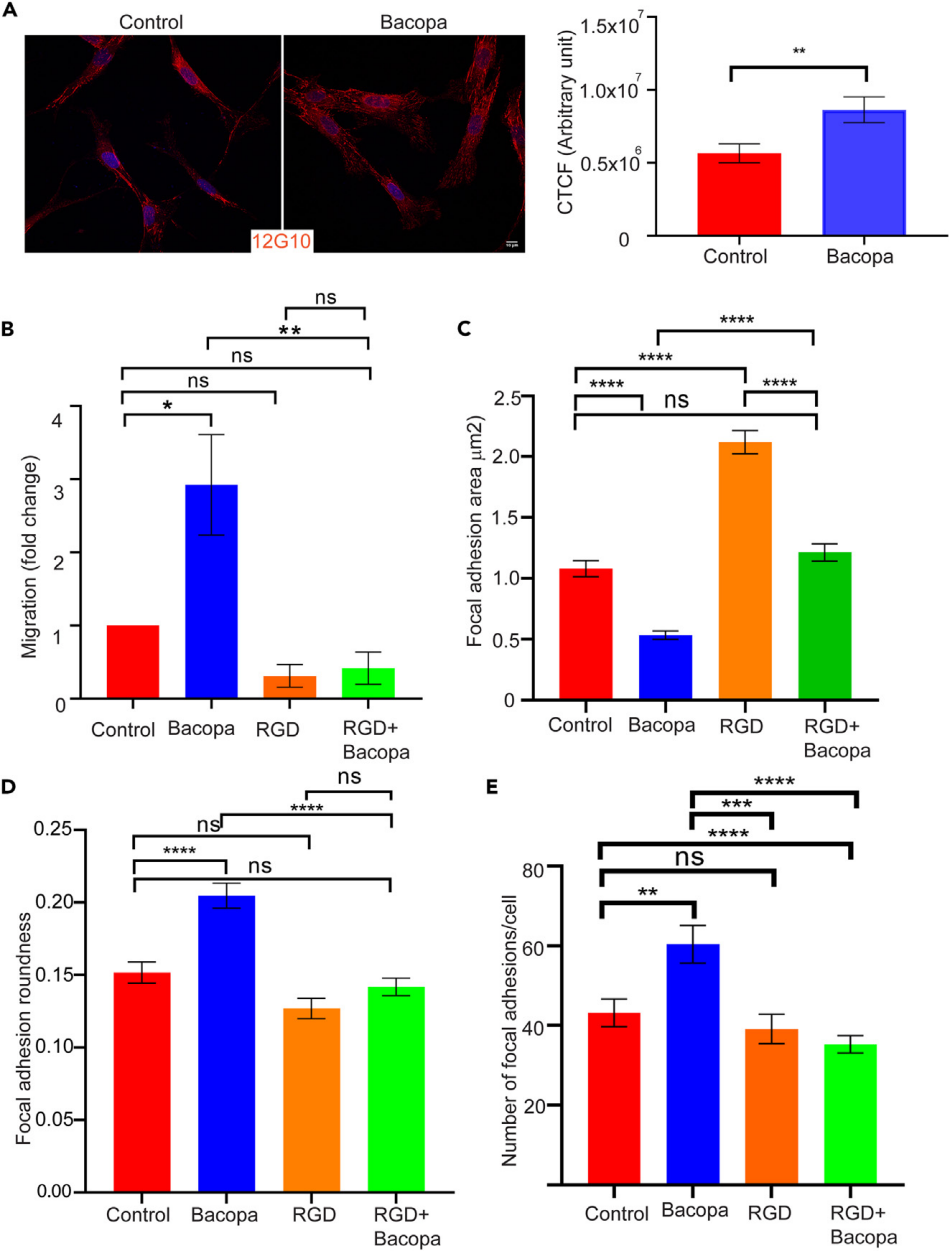
α5β1 integrin activation mediates *Bacopa monnieri* extract-induced migration (A) Immunofluorescence staining for active integrin 12G10 (red) in cells treated with *Bacopa monnieri* or vehicle control (left panel) and corrected total cellular fluorescence (CTCF; right panel). Scale bar: 10 μm. (B) Quantification of transwell migration of cells in the presence of vehicle control, *Bacopa monnieri* extract, RGD peptide, or *Bacopa monnieri* extract + RGD peptide. p values were calculated using unpaired Student’s t test. ∗p ≤ 0.05, (n = 3 Biological replicate). (C–E) Quantification of focal adhesion area (C), roundness (D), and number (E) in control (n = 67 cells), *Bacopa monnieri*-treated (n = 67 cells), RGD peptide-treated (n = 41 cells), or *Bacopa monnieri* extract + RGD peptide-treated cells (n = 41 cells) in three independent experiments. p values were calculated using one-way ANOVA followed by post hoc Tukey’s multiple comparisons test. ∗p ≤ 0.05, ∗∗p < 0.01, ∗∗∗p < 0.001, ∗∗∗∗p < 0.0001.

Given that integrins regulate the *de novo* assembly of focal adhesions^22^ and have a major impact on the migratory behavior of cells,^23^^,^^24^ we assessed whether *Bacopa monnieri* extract regulates these processes at the level of integrin receptors. We focused on integrin α5β1, which is found on human dermal fibroblasts and is known to modulate their migration.^24^ The status of α5β1 integrin activation was assessed by immunostaining the *Bacopa monnieri* extract-treated cells with an active integrin antibody (12G10), which detects the integrin only when it is in its active conformation. Corrected total cell fluorescence (CTCF) analysis by ImageJ revealed that *Bacopa monnieri* extract increased the activation of integrin by ∼50% compared to the vehicle control (Figure 3A). To investigate whether the *Bacopa monnieri*-mediated activation of α5β1 is responsible for the enhanced cellular migration, we inhibited the integrin with the arginine-glycine-aspartic acid (RGD) peptide that competitively binds to their ECM binding site.^25^ Pre-treatment of cells with a high concentration (500 μM) of RGD peptide was done followed by a transwell migration assay in the presence of *Bacopa monnieri* extract in the lower chamber. Interestingly, it was observed that the addition of RGD peptide effectively blocked the *Bacopa monnieri* extract-induced chemotaxis (Figure 3B). To test if inhibition of integrin signaling by RGD could inhibit the decrease in the size of focal adhesions caused by *Bacopa monnieri*, human dermal fibroblasts were treated with extract in the presence of 500 μM of the RGD peptide. Cells were immunostained for vinculin and analyzed for the effect on focal adhesion size. It was observed that RGD peptide increased the size of focal adhesions significantly compared to the vehicle control. Interestingly, inhibition of integrin activation in *Bacopa monnieri* extract-treated cells blocked the reduction in the size of focal adhesions (Figure 3C). Inhibition of integrin activity could also reduce the roundness of focal adhesions in *Bacopa monnieri* extract-treated fibroblasts (Figure 3D). Additionally, the RGD peptide significantly inhibits the increase in the number of focal adhesions per cell compared to *Bacopa monnieri* extract alone (Figure 3E). Taken together it was observed that *Bacopa monnieri* extract increases fibroblast cell migration by activating integrin signaling.

### *Bacopa monnieri* extract activates focal adhesion kinase

**Figure 4:**
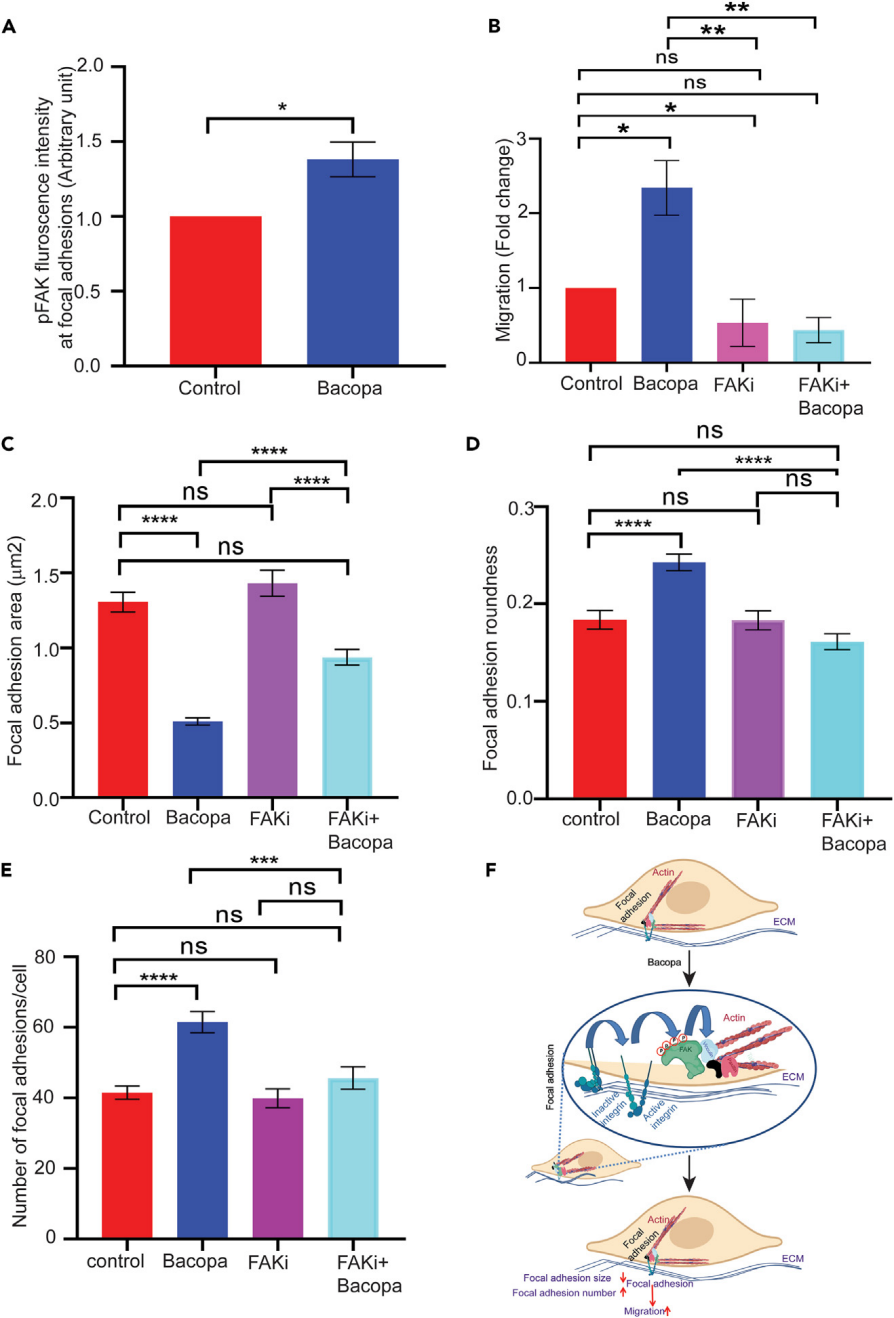
Focal adhesion kinase activation mediates *Bacopa monnieri* extract induced migration (A) Fluorescence intensity of phospho FAK in human dermal fibroblasts treated with *Bacopa monnieri extract* or vehicle control. (B) Quantification of transwell migration of cells in the presence of vehicle control, *Bacopa monnieri* extract, FAK inhibitor, or *Bacopa monnieri* extract + FAK inhibitor. p values were calculated using unpaired Student’s t test. ∗p ≤ 0.05, (n = 3 biological replicate). (C–E) Quantification of focal adhesion area (C), roundness (D), and number (E) in control (n = 41 cells), *Bacopa monnieri*-treated (n = 67 cells), FAK inhibitor-treated (n = 41 cells), or FAK inhibitor + *Bacopa monnieri* extract-treated cells (n = 61 cells) in three independent experiments. p values were calculated using one-way ANOVA followed by post hoc Tukey’s multiple comparisons test. ∗p ≤ 0.05, ∗∗p < 0.01, ∗∗∗p < 0.001, ∗∗∗∗p < 0.0001. (F) Model.

One mechanism by which integrin signaling mediates migration is through focal adhesion kinase (FAK).^24^ The status of FAK activation was assessed by immunostaining the *Bacopa monnieri*-treated cells with pFAK antibody which selectively detects the phosphorylated (active) form of FAK (Figure S2D). Interestingly it was observed that *Bacopa monnieri* extract increased the phosphorylation of FAK by ∼30% (Figure 4A). To investigate if FAK activity mediates *Bacopa monnieri* extract-induced cellular migration, we inhibited the FAK phosphorylation with 5 μM of the inhibitor PF-573228. Interestingly, it was observed that the addition of FAK inhibitor blocked the *Bacopa monnieri* extract induced transwell migration to control levels (Figure 4B). Likewise, we investigated whether inhibition of FAK signaling can abrogate the reduction in the size of focal adhesions caused by *Bacopa monnieri* extract. Human dermal fibroblasts were treated with extract in the presence of FAK inhibitor and focal adhesions were visualized with an antibody against vinculin. Interestingly, while FAK inhibitor alone did not affect focal adhesion size relative to control, the inhibitor restored the focal adhesions to normal size in *Bacopa monnieri* extract-treated cells (Figure 4C). Similarly, inhibition of FAK phosphorylation inhibits the increased roundness of focal adhesions in *Bacopa monnieri* extract-treated fibroblasts (Figure 4D). In addition, the restoration of focal adhesion morphology by FAK inhibitor also resulted in the normalization of focal adhesion number in *Bacopa monnieri* extract-treated cells (Figure 4E).

## Discussion

Altogether the data presented suggest a mechanism by which *Bacopa monnieri* extract can induce the migration of dermal fibroblast. Primary human dermal fibroblasts exposed to *Bacopa monnieri* extract can activate integrin α5β1 which subsequently leads to the activation of FAK. The active kinase leads to a decrease in the size and increase in the number of focal adhesions (Figure 4F). These data are in line with reports that smaller focal adhesions correlate with a higher migratory capacity of the cell.^20^^,^^26^ Consequently, this model partly explains the mechanism by which *Bacopa monnieri* extract can accelerate wound healing that was observed *in vivo*. It has previously been shown in the *in-vivo* model of excisional wound healing that the Bacopa extract can promote re-epithelialization and collagen production^13^ suggesting that this extract might work in all phases of the wound healing program. However, the mechanism of action of *Bacopa monnieri* extract was not elucidated. In addition, the study was based on the systemic effect of Bacopa so the direct effects of the extract were not known. Our *in vitro* studies demonstrate that the Bacopa extract can have a direct effect on dermal fibroblasts. By stimulating the migration of resident dermal fibroblasts, the *Bacopa monnieri* extract can drive the recruitment of these cells into the wound bed where they play important roles in regulating the tissue repair response such as inflammation, angiogenesis, and ECM production/remodeling. Deciphering the mechanism of action of *Bacopa monnieri* extract fills an important gap in the field of herbal remedies where the cellular processes modulated by phytochemicals are largely undetermined. Another implication of the effect of Bacopa monnieri extract on focal adhesions is its potential impact on the mechanosensory capacity of the fibroblasts. Focal adhesion are known to be important components of the mechanotransduction machinery,^27^^,^^28^^,^^29^ and can thus play a role of the response of cells to the different tensions of the extracellular milieu. However, an outstanding issue is the identification of the active principle of the extract responsible for the stimulation of fibroblast migration. We have tested some of the purified molecules from *Bacopa monnieri* extract such as bacoside A which is standard mixture of bacoside A3, bacoside II, jujubigenin, and bacopasaponin C. Unlike the crude extract of Bacopa, this mixture of purified molecules had no effect on accelerating the cell migration in fibroblast (Figure S2E). This indicates that additional investigation into the active components of the *Bacopa monnieri* extract is required to further elucidate the mechanism by which it stimulates the cell migration.

Though the use of herbal remedies generally lacks an in-depth mechanistic understanding, this method to promote physiological processes or correct pathological scenarios has various advantages over synthetic drugs. The latter has several drawbacks such as higher side effects and cost compared to herbal medicine.^30^ Moreover, herbal remedies are generally more widely available and non-toxic for therapeutic uses and are based on a more holistic approach rather than targeting specific symptoms of a disease.

The application of *Bacopa monnieri* extract in the wound healing context illustrates this holistic approach to restoring homeostasis. There are multiple reasons for the impairment of wound healing which may include local, systemic factors, and reduced tissue growth factors. Local factors comprise tissue maceration, foreign bodies, biofilm, hypoxia, ischemia, and wound infection. Systemic factors comprise diabetes, malnutrition, and other chronic organ diseases. In general, it is not possible to remove these factors completely even in good clinical practices.^6^ In addition to local and systemic factors, reduced levels of tissue-related growth factors increased proteolytic enzymes, and increased inflammatory mediators, also participate in a delayed wound healing program.^31^ As a result of the multi-tiered defects that lead to chronic, non-healing wounds many processes within the wound healing program are perturbed. We have now shown that the pro-migratory effect of *Bacopa monnieri* extract on human dermal fibroblasts complements the reported holistic effect of this herbal remedy that also increases the wound closure rate in rats, counteracts free radicals and reduces inflammation.

### Limitations of the study

The main limitation of the study is that we have shown the effect and mechanism of action elicited by the Bacopa extract, but what remains unanswered is the active principle in the extract responsible for this activity. In addition, the *in vitro* results should be validated *in vivo*; however, due to the source of funding, animal studies were prohibited due to ethical concerns. Though the effect of Bacopa on different phenotypes such as migration, focal adhesion changes, integrin activation, and FAK activation was documented, it is important to include a comparative benchmark with known inducers of these phenotypes.

## STAR★Methods

### Key resources table

**Table:** 

REAGENT or RESOURCE	SOURCE	IDENTIFIER
**Antibodies**		

Anti-integrin β1 antibody (12G10)	Abcam	Cat. #ab30394; RRID:AB_775726
phospho-FAK (Y397)	Thermo Fisher	Cat. #44624g; RRID: AB_2533701
Goat anti Rat Alexa Fluor 488	Invitrogen	Cat.# A11006; RRID: AB_2534074
Goat anti Rat Alexa Fluor 568	Invitrogen	Cat. # A11077; RRID: AB_2534121
Anti-Vinculin	Sigma Aldrich	Cat. #V9264

**Chemicals, peptides, and recombinant proteins**		

Methanol	SPECTROCHEM PVT LTD	71301
RGD peptide	Santacruz Biotechnology Inc (Bibootech)	Cat.SC-201176
FAK inhibitor- PF-573228	Sigma Aldrich	PZ0117
DMEM media	VASA SCIENTIFIC (HIMEDIA)	AT066-10X1L
Cell Proliferation Reagent WST-1 (2X8mL)	Roche	5015944001
PrimeScript cDNA synthesis kit	Takara	Cat.#2680A

**Deposited data**		

RNA sequencing raw data	NCBI data	PRJNA939001

**Experimental models: Cell lines**		

Human adult dermal fibroblasts	Lonza	Cat. Lonza CC-2511

**Oligonucleotides**		

PTGS2 forward primer TCCAATGACTCCCAGTCTGAGGA,	This paper	NA
PTGS2 reverse primer TCAAAGGTCAGCCTGTTTAC	This Paper	NA
KIT forward primer TGTGTTGTCACCCAAGAGATT	This Paper	NA
KIT reverse primer CAATGAAGTGCCCCTGAAG	This Paper	NA
ARHGAP22 forward primer TACAGGGGCTGGTCACTGAG	This Paper	NA
ARHGAP22 reverse primer GTTCCGCAGCTTTATTTCCA	This Paper	NA
NEDD9 forward primer GCCTCTAGAAGCAAGTCCGC	This Paper	NA
NEDD9 reverse primer GGGAGGTGACAGCTAGTCCT.	This Paper	NA

**Software and algorithms**		

ImageJ software (Fiji)	Schneider et, al.^32^	https://imagej.nih.gov/ij/
Prism version 7.0	GraphPad	https://www.graphpad.com/

### Resource availability

#### Lead contact

Lead Contact, Colin Jamora (chs1927b@gmail.com).

#### Material availability

This study did not generate new unique reagents. Further information and requests for resources and reagents should be directed to and will be fulfilled by the lead contact.

#### Data and code availability

The raw data for RNA sequencing is uploaded on the NCBI database with accession number PRJNA939001 Databse: https://www.ncbi.nlm.nih.gov/bioproject/PRJNA939001/. Data reported in this paper will be shared by the lead contact upon request. This paper does not report original code. Any additional information required to reanalyze the data reported in this paper is available from the lead contact upon request.

### Experimental model and study participant details

#### Primary cell cultures

Human adult dermal fibroblasts (Lonza CC-2511) were cultured in DMEM media with (10% FBS, Pen-Strep, NEAA, and sodium pyruvate) in 5% CO2. All the experiments were performed with 3 biological replicates.

### Method details

#### Test substance

*Bacopa monnieri* extract was procured from Natural Remedies Pvt. Ltd, Bangalore under the brand name BacoMind. It has the following bioactive constituents viz., bacoside A3 (4.5% w/w), bacopaside I (5.1% w/w), bacopaside II (0.5% w/w), jujubogenin isomer of bacopasaponin C (5.8% w/w), bacopasaponin C (4.8% w/w), bacopaside-I (5.1%), bacosine (2.0% w/w), luteolin (0.47% w/w), apigenin (0.21% w/w), β−sitosterol D glucosides (0.36%) quantitated by HPTLC. Other bacosides (12.9%) was quantified by HPLC using bacoside A as a standard (Table S1). The structures of these chemicals have been previously reported.^33^

#### RNA extraction, cDNA preparation, and real-time PCR

Human adult dermal fibroblasts were lysed in 1 mL Trizol reagent (Invitrogen, 10296028), and RNA was isolated according to the manufacturer’s protocol. cDNA was synthesized by PrimeScript cDNA synthesis kit (Takara, Cat No: 2680A) according to the manufacturer’s protocol. Quantitative PCR (qPCR) was carried out using Power SYBR Mix (Applied Biosystems, Thermo Fisher Scientific, Cat No: A25742) in a Bio-Rad CFX384 machine. GAPDH or TBP expression was used for normalization. Primers used for qPCR are listed below. PTGS2 forward primer) TCCAATGACTCCCAGTCTGAGGA, PTGS2 reverse primer TCAAAGGTCAGCCTGTTTAC, KIT forward primer TGTGTTGTCACCCAAGAGATT, KIT reverse primer CAATGAAGTGCCCCTGAAG, ARHGAP22 forward primer TACAGGGGCTGGTCACTGAG, ARHGAP22 reverse primer GTTCCGCAGCTTTATTTCCA, NEDD9 forward primer GCCTCTAGAAGCAAGTCCGC, NEDD9 reverse primer GGGAGGTGACAGCTAGTCCT.

#### RNA Seq and data analysis

Adult human dermal fibroblasts were treated with vehicle control (water) or *Bacopa monnieri* (5 μg/mL) for 12 h and RNA was isolated by Trizol method followed by phase separation with chloroform. Finally the RNA was washed with 70% ethanol. Further the dried RNA pellet was dissolved in distilled water and it was given for RNA sequencing. RNA sequencing was performed by MedGenome (Bangalore, India) and ∼45–55 million 150 bp paired-end reads were derived from three replicates of each treatment. The analysis was performed by using standard RNA Seq analysis pipeline. FASTQC tool (https://www.bioinformatics.babraham.ac.uk/projects/fastqc/) was employed for the QC of the raw sequencing reads. Low-quality reads at both ends were trimmed using “FASTX-TRIMMER” by using the parameters as “-f 11 -L 130” (http://hannonlab.cshl.edu/fastx_toolkit/index.html). Only the good-quality trimmed reads (120bp∗2) were aligned with the human reference genome (hg38, UCSC version) using “HISAT2.”.^34^ The “Count” matrix was created using the “BAM” outputs using “HTSeq-count”.^35^ The differentially expressed genes were identified from the count matrix by using “DESeq2” R package.^35^ Moreover, the significantly differentially expressed genes were used for Gene Ontology enrichment analysis using “DAVID”.^36^

#### Transwell migration assay

Transwells with 8 μm pore size (Corning, Cat. #3422) was used for transwell migration assay as described previously.^37^ In short 75000 fibroblast were seeded on the transwell chamber. After 12 h post seeding the cells were treated Bacopa extract with 10 μg/mL of *Bacopa monnieri* or vehicle control was added to the bottom compartment. After 12 h post treatment t he number of migrated cells on the lower compartment were fixed with 4% PFA for 5 min and stained with 2.5% crystal violet dye. The stained cells were imaged with 10X objective and cells were counted in three different fields.

#### Cell proliferation assay

Human adult dermal fibroblasts were seeded at a density of 20000 cells per well in 24 well plates. Cells were cultured in 10% serum for 24 h, followed by treatment with vehicle control or *Bacopa monnieri* extract 5 μg/mL in serum-free media. Cells were trypsinized and counted by using a hemocytometer on day 1, 2, 3.

#### Cell viability assay

1000 human adult dermal fibroblasts were seeded per well of a 96 well plates and were allowed to attached completely for 24 h in complete DMEM media. Post 24 h cells were treated with vehicle control or *Bacopa monnieri* extract at doses of 5, 10, 15, 20, 25, and 30 μg/mL for 72 h. The culture media was removed and cells viability was assessed using a 10% WST reagent (Roche, Cat. #5015944001) by incubating the cells for 2 h and measuring the O.D. at 450nm.

#### Immunofluorescence

For staining, cells were fixed with 4% paraformaldehyde (PFA) (Fisher Scientific, Cat. #50-980-487) on coverslips. Anti-integrin β1 antibody (12G10) (Abcam Cat. #ab30394) was used at a dilution of 1:200. For phospho-FAK (Y397) (Thermo Fisher, Cat. #44624g) staining, cells on the coverslip were fixed with chilled methanol at −20°C for 5 min and incubated with antibody at a dilution of 1:100. Alexa Fluor 488 or Alexa Fluor 568–labeled secondary antibodies (Jackson Immuno Research) was used at a dilution of 1:200. Fluorescence intensity for anti-integrin β1 antibody (12G10) and pFAK was quantitated using ImageJ with the following formula: CTCF = Integrated Density – (area of selected cell x mean fluorescence). The size, shape, and number of focal adhesions were calculated by ImageJ software (National Institutes of Health, Bethesda, USA) by using the “Manual” freehand tool.^32^

#### Scratch migration assay

Migration in a scratch assay was performed as described.^38^ Briefly, 50000 cells were seeded in each well in 24 well plates. The cells were grown to a confluent monolayer in complete DMEM media The cells were then washed with PBS and a “wound” was introduced by scratching the plate with a sterile 1mL pipette tip. The debris was then removed by washing the cells with PBS. The cells were treated with vehicle control or 5 μg/mL *Bacopa monnieri* extract. The scratch closure was analyzed by imaging the scratch daily until the scratch was closed. The open area of the scratch was analyzed by ImageJ freehand tool. The scratch closure was plotted as % open wound.

#### Random migration assay

5000 fibroblasts were added to each well of a 24 well dish and allowed to adhere for 24 h in complete media. Cells were then treated with vehicle control or 5 μg/mL *Bacopa monnieri* extract in serum free media. Live cell imaging was performed with a phase-contrast microscope setting on IX 83 microscope with a motorized stage. 5 different fields were imaged at 15 min time intervals for 12 h. Single cells were tracked by tracking the nucleus of the individual cell by ImageJ software using the “Manual Tracking” plugin (Fabrice Cordelières, Institut Curie, Orsay, France). Each experiment was repeated three times independently. Further analysis for distance, velocity, and directionality was performed by using the Chemotaxis and Migration Tool V2.0 from Ibidi. At least 50 cells were analyzed per condition.

### Quantification and statistical analysis

A comparison of two groups to analyze effects on human adult dermal fibroblasts was made using the paired Student’s *t* test and 1-way ANOVA followed by post hoc Tukey’s multiple comparisons test. GraphPad Prism 5.02 was used for all the statistical analysis for all the experiments. Graphs represent mean ± SEM. p values less than 0.05 were considered to be significant.
