# Supplementary material for: Bacopa monnieri phytochemicals regulate fibroblast cell migration via modulation of focal adhesions
Source: iScience. 2024 Mar 11;27(4):109489. doi: 10.1016/j.isci.2024.109489 (PMC10981128; doi:10.1016/j.isci.2024.109489)
Supplement: Document S1. Figures S1, S2 and Table S1 [file mmc1.pdf]

iScience, Volume *27*

Supplemental information

Bacopa monnieri phytochemicals regulate fibroblast cell migration via modulation of focal adhesions

Ravindra K. Zirmire, Dyuti Saha, Rakesh Dey, Habibu Tanimu, Rania Zaarour, Deborah Bird, Prakash Cherian, Isha Rana, Nita Roy, Aryasekhar Sanyal, Namita Misra, and Colin Jamora

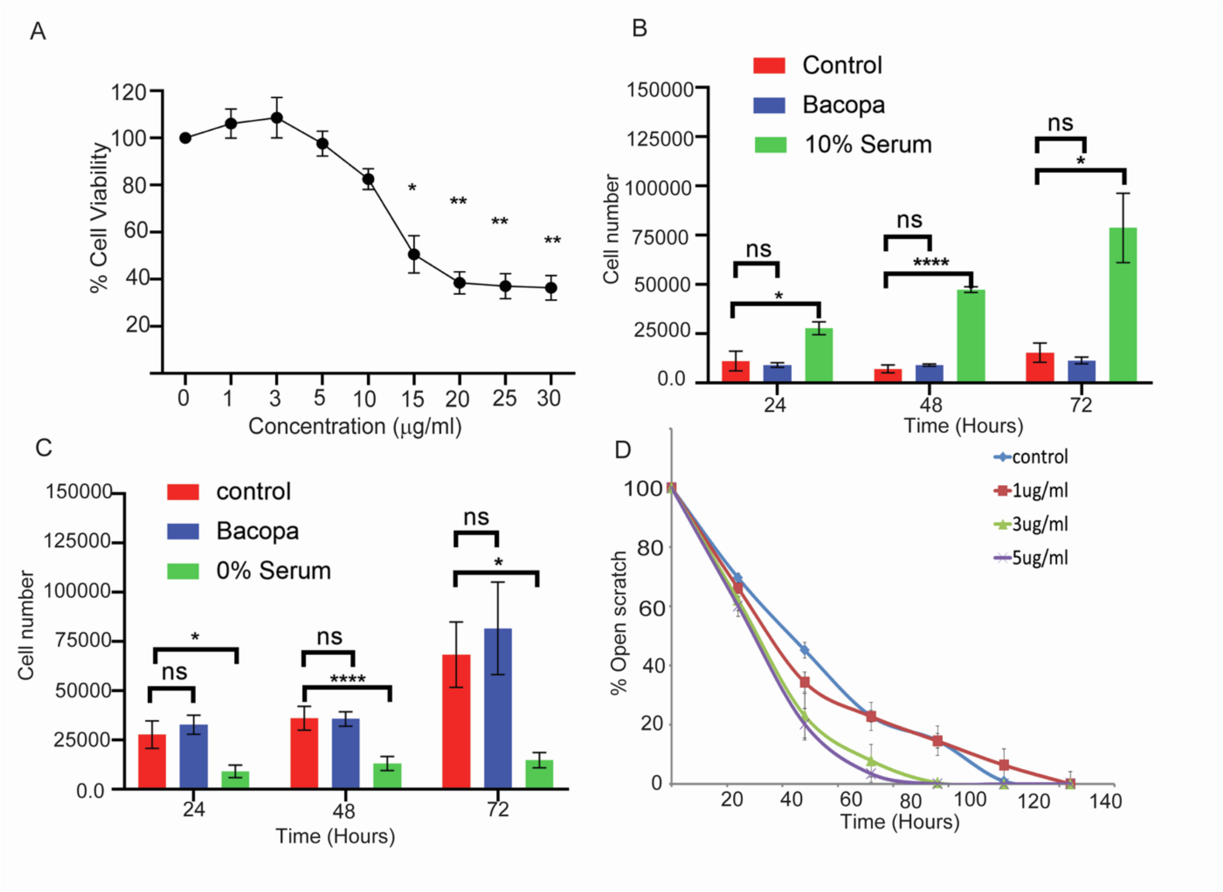

Supplementary Figure 1. *Bacopa monnieri* extract does not affect cell proliferation rate,related to figure 1. A) Maximum tolerable dose was determined by WST assay. B) Proliferation assay with WST after treating the cells with *Bacopa monnieri* extract or vehicle control. C) Proliferation inhibition assay with WST after treating the cells with *Bacopa monnieri* extract or vehicle control. For figures A-C, p values were calculated using one way ANOVA followed by Tukey's test. *$p<=0.05$, **$p<0.01$, ***$p<0.001$, three independent biological replicates D) Scratch migration of cells in the presence of Bacopa or vehicle control (n=3 biological replicates).

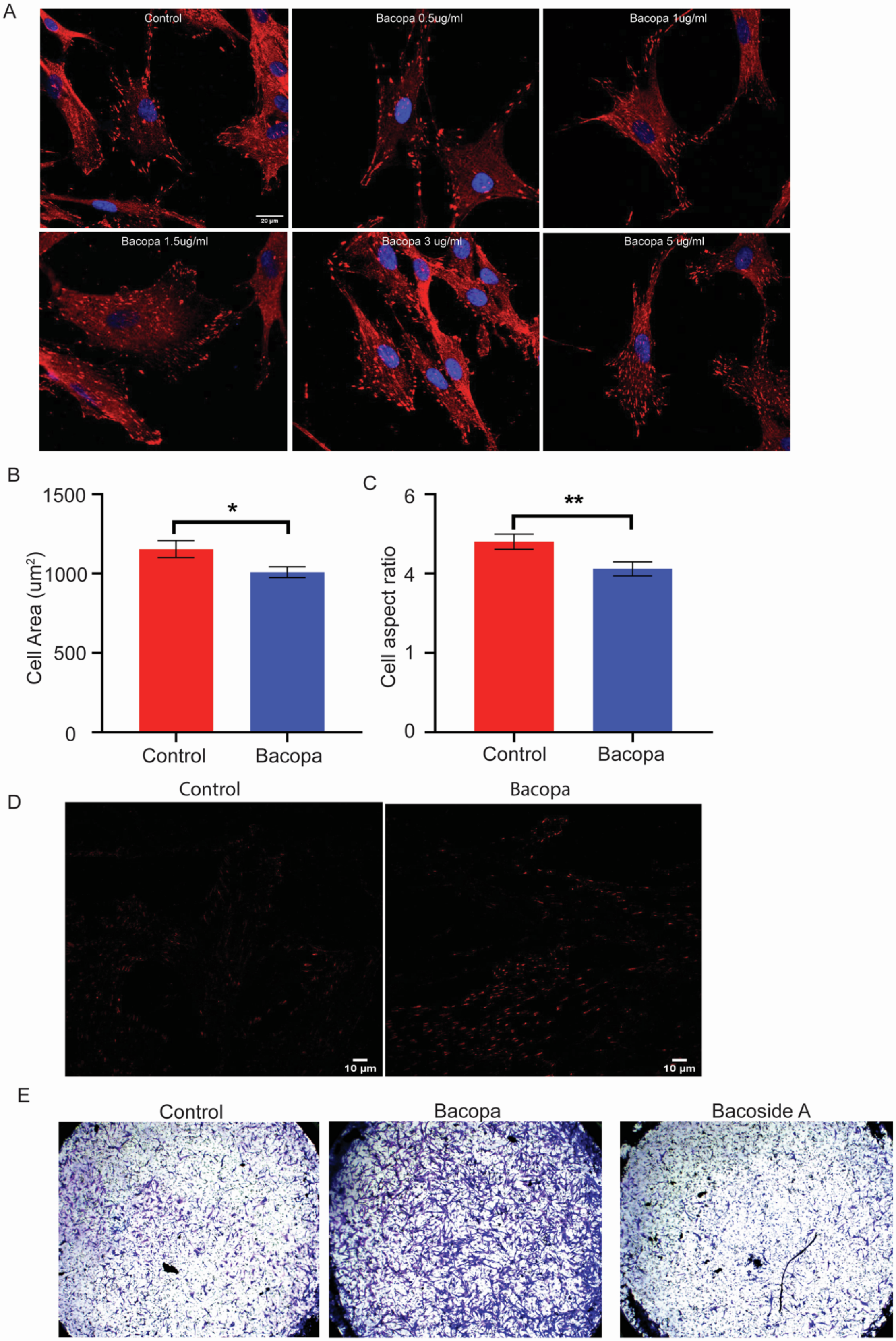
A
Control
Bacopa 0.5ug/ml
Bacopa 1ug/ml
20 µm
Bacopa 1.5ug/ml
Bacopa 3 ug/ml
Bacopa 5 ug/ml
B
Cell Area (um2)
1500
1000
500
0
*
Control
Bacopa
C
Cell aspect ratio
6
4
1
0
**
Control
Bacopa
D
Control
Bacopa
10 µm
10 µm
E
Control
Bacopa
Bacoside A

Supplementary Figure 2. Bacopa monnieri extract activates focal adhesion kinase related to figure 2,4 and discussion A) Immunofluorescence staining for focal adhesion marker vinculin. B) Cell area and C) shape of fibroblasts treated with *Bacopa monnieri* extract. D) Immunofluorescence staining for p-FAK in the cells treated with Bacopa monnieri extract of vehicle control. Scale bar 10μ m. E) Transwell migration of fibroblasts treated with Bacoside A which is standard mix of purified bacoside A3, Bacoside II, jujubigenin and bacopasaponin C at 1μg/ml.

Chemical	%W/W	Method of Analysis
Bacoside -A3	4.5	HPLC
Bacopaside-II	0.5	HPLC
Bacopaside -X (jujubogenin isomer of bacopasaponin C)	5.8	HPLC
Bacopasaponin C	4.8	HPLC
Bacopaside -I	5.1	HPLC
Luteolin	0.47	HPLC
Apigenin	0.21	HPLC
Bacosine	2	HPLC
Beta sitosterol D glucosides	0.36	HPTLC
other bacosides	12.9	HPLC (as bacoside A)

Supplementary table: HPLC quantification of the different chemicals present in the *Bacopa monnieri* extract, related to method details test substance.
